# Exploring active ageing in a community-based living environment: an ethnographic study in the Western Norway context

**DOI:** 10.3389/fpubh.2024.1380922

**Published:** 2024-04-30

**Authors:** Elise Førsund, Juan Carlos Torrado Vidal, Stein Erik Fæø, Haakon Reithe, Monica Patrascu, Bettina S. Husebo

**Affiliations:** ^1^Department of Global Public Health and Primary Care, Faculty of Medicine, Centre for Elderly and Nursing Home Medicine (SEFAS), University of Bergen, Bergen, Norway; ^2^Norwegian Computing Centre, Oslo, Norway; ^3^Department of Nursing, Faculty of Health Studies, VID Specialized University, Bergen, Norway; ^4^Neuro-SysMed Centre, University of Bergen, Bergen, Norway

**Keywords:** active ageing, community-based environments, senior housing, age-friendly communities, loneliness, ethnography, reflexive thematic analysis, Norway

## Abstract

**Background:**

Age-friendly environments intend to promote active ageing by facilitating social, mental, and physical participation. This could potentially delay the onset of chronic complex conditions, enabling people to live longer independently at home, and prevent loneliness. This study investigates a community-based living environment in Norway called Helgetun and aims to explore how it can facilitate active ageing.

**Method:**

We chose an ethnographic approach consisting of observation, informal conversations, and in-depth semi-structured interviews with 15 residents (11 female, 4 male, ages 62–84). We analysed the data using reflexive thematic analysis.

**Result:**

We developed three themes on facilitating active ageing in this living environment: maintaining self-identity, experiencing growth and development, and feeling a sense of belonging. These themes were related to physical activity levels, social engagement, and overall satisfaction with the living environment. Maintaining self-identity concerned getting a new role in life as well as access to meaningful activities. Experiencing growth and development involved being exposed to new activities, learning new skills, and experiencing mastery. Lastly, feeling a sense of belonging meant feeling safe and part of a group, as well as receiving social support and help. This feeling of social connectedness and safety was reflected in their experience with the COVID-19 pandemic, in which most felt relatively unaffected, suggesting that this way of living could increase reliance among this age group.

**Conclusion:**

Having a flexible structure, adapting to the core needs and individual resources of the residents, can facilitate active ageing in community-based living environments. Our findings contribute to the growing evidence that these environments increase social and physical engagement, whilst reducing social isolation and loneliness. These findings may be particularly relevant in a Norwegian context—where older adults are less dependent on family for care—and are meant as grounding points for policymakers to reflect upon designing future senior living.

## Background

The world population is rapidly ageing due to increased life expectancy and declining birthrates (1). This causes a shift in the age-dependency ratio, which has severe implications for society, with a larger proportion of the population in need of care and fewer health-care workers to take care of them. Consequently, older adults are now expected to live at home for longer, even in countries like Norway which has a long tradition of institutionalised care. However, ageing at home may lead to social isolation, which again may lead to the feeling of loneliness. This subjective, negative feeling is caused by a discrepancy between a person’s actual and desired social needs (2). Additionally, ageing at home may result in a more sedentary lifestyle, which is associated with declining health and greater need for care (3, 4). To meet these challenges, there has been a shift in objectives towards targeting people at an earlier stage in life for better health outcomes in later years. One strategy is to develop alternative living solutions, in between ageing at home and a nursing home, which aims to support good health and well-being by promoting active ageing.

Age-friendly environments are of high scientific relevance, with the World Health Organization (WHO) declaring it as one of its main areas of action in the 10-year global action plan “United Nations Decade of Healthy Ageing (2021–2030)” (5). This action plan is a global collaboration aiming to improve the lives of older adults. It builds upon WHOs “Active Ageing” policy framework from 2002, which aimed to inform and discuss action plans to promote healthy and active ageing. The term “active ageing” is here defined as “…the process of optimizing opportunities for health, participation and security in order to enhance quality of life as people age” (6). Thus, age-friendly environments aim to facilitate social, mental, and physical participation, thereby enabling people to continue doing things they value, live dignified lives, and potentially prevent or delay the development of chronic complex conditions and functional decline (5).

Several variants of age-friendly environments have been established in recent years including community-based environments, senior housing, co-housing, and independent living environments (7). Although they vary in design and terminology, they share the same goal of supporting ageing in place and independent living. A qualitative study by Rusinovic et al. included eight co-housing communities in The Netherlands and found increased social contacts, social control, and instrumental and emotional support (8). They especially highlighted that fewer residents experienced social loneliness compared to national statistics (8). Comparable findings were described in a Finish study by Jolanki et al. including residents of a senior housing complex with focus on physical, social and safety support (9). This study found that the housing encouraged and enabled residents to be physically active and independent, whilst providing them with social activities and feeling safe. Another mixed-method trial from Canada used the WHO Quality of Life (QoL) survey (WHOQOL-BREF) and described a co-housing related increase in residents’ QoL (10). In summary, these senior-housing models seem to fulfil their aim of creating environments supporting well-being for older adults.

Meanwhile, cultural implications may vary between different countries. Thus, it would be of interest to gain a deeper understanding of the underlying mechanism of how comparable environments may encourage older adults in Norway to choose a more active and social lifestyle. This knowledge could potentially be implemented into existing and planned living arrangements to help them facilitate active ageing. In this study we are investigating a community-based living environment for older adults called Helgetun, located in a rural area of Western Norway. Helgetun is composed of 31 rental apartments with several shared facilities and a broad variation of arranged activities and opportunities for the residents. The housing project is the first of its kind in Norway and has a vision of creating a retirement life to look forward to by facilitating social engagement, safety, creativity, and activity. Using an ethnographic approach, we aim to explore the residents’ perception of active ageing and understand how living in a community-based environment can help facilitate it. For this purpose, we propose two main research questions:

RQ1: How does living in this community-based living environment affect social engagement and physical activity levels?

RQ2: What mechanisms in this community-based living environment are important for facilitation of active ageing?

### Method

This is an ethnographic study aiming to explore the lives of older adults living in a community-based living environment, using reflexive thematic analysis to interpret and understand how this way of living can facilitate active ageing.

## Research design

The study is part of an umbrella project called ActiveAgeing, which aims to investigate the current possibilities for enhanced activity and quality of life in healthy older adults and people with Parkinson’s disease. A method paper for the ActiveAgeing project was published in 2022 (11).

We framed this ethnographic design within an interpretivist research paradigm (12). Ethnography enables direct access to the culture and its perspectives, experiences, beliefs, and practices of the community by studying the participants in their natural habitat (13). Thus, data were set to be collected in the field by the primary researcher, EF, through a combination of observation, informal conversations, and in-depth semi-structured interviews. Individual interviews were chosen over focus groups, due to the closeness and social dynamics already existing at the residency. The interview guide was developed by the ActiveAgeing research group and reviewed by a user representative (RS) associated with the group. Main topics of interviews were physical health, mental health, social dynamics, and active ageing.

### Setting and participants

The study took place in Helgetun, a senior housing project located in a rural area of Bergen, a town in Western Norway. It was founded and developed by the GC Rieber Foundation and was finished in 2019. Residents were selected by the project developers through private interviews after applying for an apartment.

The residency consists of 31 rental apartments arranged in three building blocks with common areas in between, and green areas surrounding the buildings. Some of the apartments have a window view to the nearby farm, where animals graze freely in the summertime. Each apartment is furnished and decorated – and to some degree designed—by the residents themselves, giving them a personal touch. Common facilities include a shared apartment to casually meet up for a chat and coffee and a communal building which they can book for social arrangements. At this building, a chef comes once a week to prepare a joint dinner for everyone to join. Residents can choose to participate in a variety of activities being arranged at the residency by the residents themselves, including choir, gymnastics, gardening, bridge, hiking, knitting and reading groups. They also have the opportunity to volunteer at the nearby farm or kindergarten. Due to the rural location of Helgetun, public transport services are somewhat limited, resulting in most residents owning a car and offering each other a ride when needed.

Recruitment to the study was done using a voluntary response sampling strategy, in which all residents were given information about the project and those interested in joining signed up afterwards. Information was provided by e-mail, brochures, and two presentations held at the residency by the research group. The only inclusion criteria to join the study was residency at Helgetun.

### User involvement

Two user representatives were involved in the study: one representative associated with the UiB research group (RS) and one representative from Helgetun (KO). They were included in several steps of the research process, from the study design to the data collection phase, ensuring that the voices of the participants were heard and incorporated. Four presentations of the project were held before conducting the study, two at the University and two at Helgetun, in which the user representatives were present. RS also reviewed and revised the planned interview guide.

### Author positionality and reflexivity

The research team responsible for designing and conducting the study consists of a medical doctor (BSH), a molecular biologist and civil engineer (EF), a computer scientist (JCT), a registered nurse (SEF), a system engineer (MP) and a neurophysiology scientist (HR). This multidisciplinary team of researchers offered various perspectives and a range of expertise throughout the study.

All data were collected and analysed by me, EF, the principal researcher. To facilitate the ethnographic perspective of this paper, I will use the first person singular to state my positionality and present the results and the discussion. I am a white, female, Norwegian PhD-candidate in my late twenties. My educational background is in molecular biology, in addition to civil engineering, with a focus on building design. Before starting the study, my experience with ageing was mainly related to my relationship with my grandparents, in addition to a more theoretical knowledge from my master thesis where I studied ageing on a molecular level. My general perception of senior living was that it mainly consisted of a sedentary lifestyle at home in the family house, until eventually needing to relocate to a nursing home due to increased care requirements. Thus, when reading about Helgetun, I found this to be very innovative and inspiring, and very different from my preconceptions of senior living. Nevertheless, I expected the people who lived there to be a relatively homogeneous group of particularly outgoing, physically active, and adventuring individuals. These assumptions were also based on the core philosophy of the housing project being an active and social place to age, and because they applied to move there even though the concept was new and explorative.

### Data collection

Data were collected between December 2021 and March 2022 by me, EF. Observational data were collected throughout this entire period. For the informal conversations and interview data, the 15 participants were separated into four groups and the data collection lasted two weeks per group. During these weeks, I visited them separately at the residency every second day, for informal conversations. These visits lasted between 15–50 min. At the end of the two weeks, individual semi-structured interviews were arranged at their apartments. Interviews were audio-recorded (Olympus WS-853) and lasted between 30–60 min. The first group consisted of only two participants and functioned as a pilot to test the data collection process and interview guide. No alterations were made after the first group.

A friendly relationship between me and the participant was established at the point of the interviews, mainly due to the regular visits prior to the interviews, where we both shared from our life in a natural and unstructured setting. This relationship facilitated a relaxed and safe atmosphere at the time of the interviews, in which the participants seemed comfortable sharing information about their life situation, thus providing rich data. I describe potential biases of this closeness in the limitations of this study. An interview topic guide is presented in Table 1.

**Table 1:** Interview topics.

**1. How would you describe your current physical health?** *Prompts:* What type of activities do you do? Do you feel your activity level has changed after moving to Helgetun? Do you have any physical limitations? Has the pandemic affected your activity level?
**2. How would you describe your current mental health?** *Prompts:* What activities do you do to maintain good mental health? Do you feel your mental health has changed after moving to Helgetun? Do you have any challenges regarding mental health? Has the pandemic affected your mental health?
**3. How social do you feel that you are?** *Prompts:* What social activities do you participate in? Are you satisfied with your level of social engagement? Do you participate in more social activities after moving to Helgetun? Has the pandemic affected your level of socialisation?
**4. Active ageing – what activities keep you generally active?** *Prompts:* What is your motivation for being active every day? What do you find challenging about being active? How important is being active for you? What influence does your closest have on your activity level? Are you satisfied with your level of activity in life?

### Analysis

Due to the exploratory nature of the study design and the involved role of the researcher, Braun and Clarke’s reflexive approach to thematic analysis (RTA) was deemed the most suitable analytical method (14). This approach sees the researcher’s subjectivity as a resource, something that fits naturally with ethnographic studies which are inevitably subjective. According to this methodology, subjectivity is also a necessity when studying such a complex phenomenon as ageing, which possesses major cultural differences in the way of living. Acknowledging that my positioning affected the impression and interpretation of the community was an important aspect in the context of this study. RTA also enables flexibility, making it well suited for this study which contains data from multiple data collection methods (observation, fieldnotes, interviews). The observational data were not directly analysed with RTA but shaped the coding process and my interpretation of the interview data. As the study used an exploratory and inductive approach, I went into the study with limited theoretical assumptions. During the latter steps of analysis, the preliminary findings were linked to theory, based on input from the other co-authors with relevant background in human behavior. The whole research team contributed to developing the final themes.

**Figure 1:**
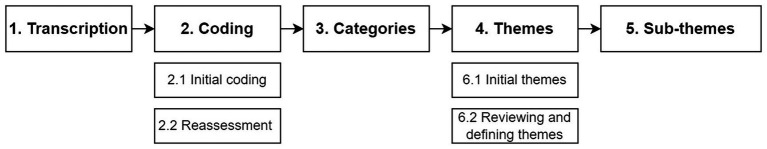
Thematic reflexive analysis steps.

An overview of the main steps of the analysis is shown in Figure 1. I transcribed all audio recordings manually as part of the content familiarization process (step 1). Analysis was conducted in the MAXQDA software 2022, using an inductive approach where I identified codes actively during the analysis of each transcript, in an iterative coding process (step 2). The initial round of coding included mostly semantic codes, labelling the transcripts descriptively and getting an overview of the content (step 2.1). Next, initial codes were organized into more latent coding-groups sharing a common meaning, defined in this context as “categories” (step 3). During this grouping process I identified new codes, and all the transcripts were reassessed with the new set of codes (step 2.2). Patterns of meaning across the dataset were then created, refined, and written up as main themes (step 4). Sub-themes were added below each main theme to structure the results section further, making it easier for the reader to follow (step 5).

## Results

### Impression of the living environment and its residents

At first sight the residency resembled a common housing association. However, in comparison to what I have experienced from other housing associations, the common areas and facilities in this residency were in regular use. In most visits, I could observe some kind of neighbour interaction: either casual conversations from the balconies or a coffee in the shared apartment. Most also attended the various activities being arranged, in particular the weekly dinner, the parcel gardening group and strength group workout. There seemed to be a well-established community in which everyone knew each other and was updated on everything going on at the residency. Residents would notice when neighbours were away or needed help with something, for instance a ride to the city. This sense of fellowship was beyond what I expected from a usual neighbourhood or housing association. Most also seemed to have distinct roles within the community, and smaller groups had emerged within the larger group based on similar personality types and interests. The location of the apartments also seemed to be a contributing factor to these social dynamics, in which residents sharing more natural meeting arenas tended to have more insight into each other’s life and whereabouts.

Unlike my expectations of meeting a homogeneous group of especially active and social individuals, I met a broad variety of people with different personalities and activity and socialization levels. Most surprising to me was how eager they were to learn new activities and improve their capabilities. This challenged my initial prejudices where I viewed ageing more as a degenerative process rather than an opportunity for growth and development. The social dynamics existing in the community, with role distributions, social grouping, and some social friction, was also surprising.

### Participants

Among the 31 residents living at the residency, 15 chose to participate in this study, all of which had lived there more or less from the beginning in 2019. They ranged in age between 62–84 years, and most of them were women living alone. Demographic data regarding gender, age, marital status, education level and financial status are presented in Table 2. All 15 participants completed the study.

**Table 2:** Participant characteristics.

Age	Participants (*N* = 15)
60–64	1
65–69	3
70–74	7
> = 75	4
**Gender**	
Man	4
Woman	11
Other	
**Education level**	
Primary school	1
High School	4
Higher education	10
**Marital status**	
Married/living with partner	4
Divorced	7
Widowed	1
Never married	3
**Self-perceived financial status**	
Very good	
Good	10
Average	5
Bad	
Very bad	

### Coding system and themes

**Figure 2:**
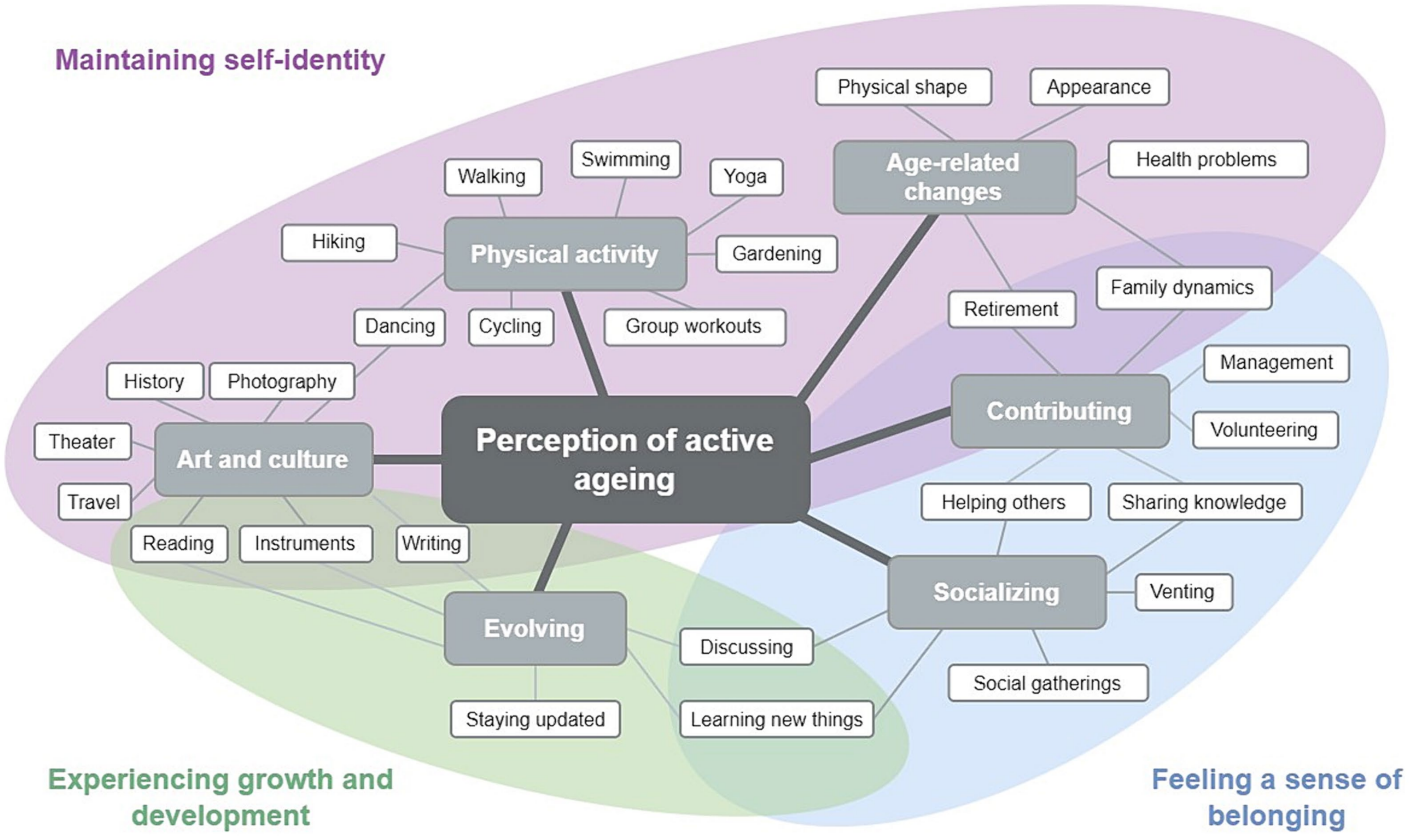
Reflexive thematic analysis map.

To understand how the living environment facilitated active ageing, we conducted in-depth interviews on participants’ thoughts on activities important for them to maintain their physical shape, mental health, social engagement and remain active into old age. Their answers were inductively organized into 6 coding categories: art and culture, physical activity, age-related changes, contributing, socializing, and evolving (Figure 2). Based on these categories, we developed three themes including their need for (1) maintaining self-identity, (2) feeling a sense of belonging, and (3) experiencing growth and development. However, these three themes were not mutually exclusive. An example of how these themes intertwine and affect the life of a participant is illustrated in the case study of “Anna” (Table 3).

**Table 3:** Participant case study.

The gardener
The following case demonstrates various ways the living environment affected the life of a resident, across the themes. Anna is 74 years old and had previously worked as a gardener. Before moving to Helgetun, she did her gardening work alone in a large garden out in the countryside. This isolated way of working, combined with increasing back pains, caused her to eventually put away all her gardening work. She even gave away all her gardening equipment, thinking she would never use them again. But after moving to Helgetun and taking part in the parcel garden group, she experienced a renewed interest in gardening, mainly due to the social engagement and interest in the activity. *Initially I had put away all the gardening work, but here the interest blossomed again! There was such intense interest in it, and it was very joyful. Completely different from what I previously did, walking alone in a large garden out in the countryside*. Regarding her physical barriers, the weekly group workouts arranged at the residency helped strengthen her back, making her more capable of performing the more physical aspects of gardening, like carrying stones and digging ditches. *It has helped me a lot that we have regular gymnastics there once a week, which has strengthened my back. So, when I have been carrying stone and such, I have managed to do it. I am very pleased*. Anna’s story is an excellent example of how the living environment can increase the well-being of older adults, by enabling the three themes: maintaining self-identity, experiencing growth and development, and feeling a sense of belonging: **Maintaining self-identity:** anna got the opportunity to practice an activity meaningful to her, by having access to a parcel garden, a gardening group, equipment, and a gym with a trainer. **Experiencing growth and development:** by doing gardening in a group, Anna was able to share knowledge and learn new things from the other members, allowing her to expand her competence within the field. **Feeling a sense of belonging:** working together as a team changed gardening for Anna, making the activity more social, joyful, and meaningful compared to what she had experienced in the past.

**Figure 3:**
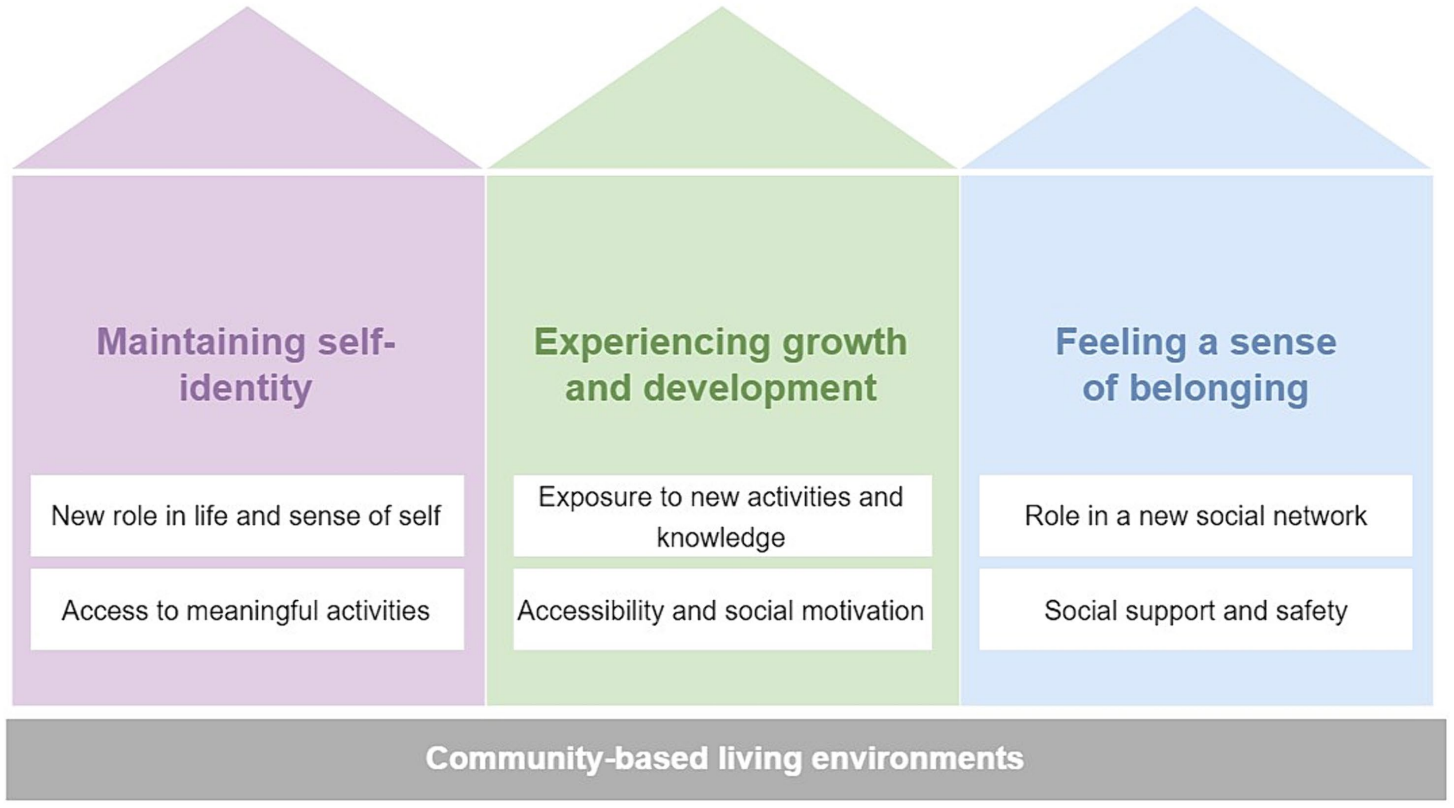
Themes and sub-themes of factors facilitating active ageing in a community-based living environment.

Each main theme was then divided into sub-themes that describe the various ways the living environment enabled the main themes. Organization of the main themes and their sub-themes are shown in Figure 3. Each theme is described in detail in the next section.

### Theme 1: maintaining self-identity

Self-identity is understood here as the sphere around which individuals project their sense of self. This sphere is composed of their personality, hobbies, interests, physical attributes, and social roles. Two sub-themes describing how living in a community-based environment affected their self-identity were developed: new role in life and sense of self, and access to meaningful activities.

#### New role in life and sense of self

For many, ageing seemed to cause a shift in how they perceived themselves and their role in life. This was demonstrated by how many tended to focus and elaborate on age-related changes, including bodily changes, changes in occupational status and changes in their role in the family. Regarding bodily changes, this was expressed by not being able to carry out physical activities with the same performance as they previously did, as well as expressing dissatisfaction over how their physical appearance had decayed with age. As one participant pointed out:

Well, I am not 30 years old anymore, so of course I notice that. I have tried a bit of jogging, but first of all I look absolutely ridiculous (laughs), I run like an old lady! And also, I think it is harder.

However, most seemed motivated to adapt to the new reality by either improving their functional ability or by doing alternative activities, for instance by exchanging running with powerwalking or a traditional bike with an electrical one. For both purposes, the living environment helped facilitate this by arranging strength and mobility workouts with a specialized trainer, as well as providing social support and inspiration to stay active. Regarding adapting to age-related changes, one participant said:

You know, 10 years ago I would have cycled up here, without help. But that is not the reality anymore, and I just have to accept it. I take it as a challenge. I have gotten myself an electric bike now, so I am very curious to see how that goes!

Changes in their role in life were expressed by acknowledging that family dynamics had changed, and that their children now had their own lives and challenges to face. Thus, when talking about their motivation for moving to the residency, many said they wished to create meaningful lives for themselves, independent of their family. Living in this new environment, with a new social network and responsibilities, therefore provided many a new role in life. As one participant explained:

And as you get older, you become less important in relation to your children, they live their lives, and I can't demand that they should be here and take care of me at all hours of the day. So, I think now I have these people I live with here.

Some participants also adapted to the transition to retirement. For some, this transition was part of the motivation for moving to the residency. As one of them explained:

Even though I was very ready to leave work, it's easy to get the feeling that you are operating on the side of society. And it is a slightly unpleasant feeling. So, in a way, that was one of the reasons why I applied to move here.

#### Access to meaningful activities

Due to the location, facilities and variety of activities being organized at the residency, most were able to continue doing activities important to them, or even take up again activities they had enjoyed in the past. This included both social and physical activities such as hiking, gardening, choir, cycling, group workouts and book clubs. One participant that picked up on reading explained:

We have this book club, and we have read so many books! I did a lot of reading in the past. Before turning 40 I always walked around with a book, but then I noticed my whole life consisted of it, so I cut it out completely. But now I am back! Not that eager though (laugh).

Although most participants were satisfied with the opportunities provided at the residency, some felt that certain possibilities were lacking compared to where they had lived in the past. Most cases were related to the location of Helgetun, including the distance to the ocean and to the city. Some mentioned the inconvenience of traveling a long way to casually catch up with friends in the city, and another participant commented on the hurdle to attend cultural events like going to the movies, theatre, or an art museum. A few participants also longed for more philosophical and intellectual content in some of the activities and were missing an arena to discuss different aspects of life with others. One of these participants said:

I am happy with the number of activities being arranged, but I would have benefited from a bit different content in some of the activities, I have to say … I like to talk and discuss matters, for instance ethical dilemmas, I am not so good at small talk (laughs).

There seemed to be a correlation between not having access to these meaningful activities and overall satisfaction with the living environment, emphasizing the importance of being able to maintain participation in interest and activities meaningful to the individual.

### Theme 2: experiencing growth and development

This theme reflected the participants’ desire to learn new things, gain new knowledge, and improve their capabilities. The different ways the living environment enabled this growth were separated into two subthemes: exposure to new activities and knowledge, and accessibility and social motivation.

#### Exposure to new activities and knowledge

Exploring new activities and learning new skills was something many found to be valuable, fun, and unexpected. Activities were organized by the residents themselves, resulting in them reflecting the residents’ various and unique interests. Some took on the main responsibility for the activities, based on their existing expertise within the specific area and functioned as teachers and organizers. This arrangement led to the introduction of activities new for many of the residents, including folk dance, origami, and parcel gardening. In particular, there was great enthusiasm related to the parcel garden. This seemed to be the most unifying arranged activity, engaging both the ones with and without previous experience with gardening. When discussing the parcel garden, one participant said:

It functions as an adult training institution, in which the ones who know a lot teach you what to do and which fruits or vegetables to use. Completely foreign things for some. And luckily, people are willing to share, and not keep their secrets to themselves. There are some who have an expertise at a doctoral level, and others who are only just in the preparatory stage.

Some also noticed areas within the group that could benefit from some guidance and arranged group sessions to help. An example of this were various presentations held at the common house, led by professors and lecturers invited to the residency by one of the residents who had previously worked at the University. Another example was a cooking class arranged particularly for the men living there. In this class, a female resident led the course and provided the groceries and recipe, and then helped the men to prepare a meal. At the end, they all shared the meal together. The female resident leading the course explained it like this:

It was certainly nice…One of them couldn't even peel a carrot, really. And they weren't used to tasting the food. But then there were some who are almost little masters! It was really a social thing, a mix of us having fun and learning something.

#### Accessibility and social motivation

Many experienced an increase in both physical activity levels and social engagement after moving to the living environment. This increase did not seem to be related to how active and social the participants perceived themselves to be before moving. One main reason for the increase was the convenience of having group activities arranged at the residency, as well as a close-by gym and hiking opportunities. One participant explained how this accessibility caused him to be more active:

There are more things being announced here, such as the group workout, the folk dance and bridge. You can also join the hiking group in the summertime if you want. And all of this comes in addition to the things you usually did right. Here you can book yourself every day if you want.

Another reason for this increase was the encouragement from neighbours doing the same activity, serving as a source of inspiration, and providing a feeling of team spirit. Upon discussing activity levels one participant said:

I think we inspire each other up here. Firstly, there are many more people who go for walks. Also, there are two hiking groups up here, one that goes quite actively on long hikes on the mountain, and some who go on shorter hikes. And when someone is being like: "join in", you do become more active.

Regarding socialization, the convenience of having someone close by seemed to lower the threshold for being social. This could include going for a walk together, joining the arranged social activities, or just casually meeting up in the common areas. Upon discussing socialization levels, one participant said:

I think that for me it has been a big plus. I have made some good friends here. I also have friends elsewhere, it's not that, but it's very easy up here. We go on little walks together and meet in different ways. You do not have to bother with traveling to meet people.

### Theme 3: feeling a sense of belonging

Feeling a sense of belonging was a theme developed based on their emphasis on connecting with others. Two sub-themes describing how the living environment affected their sense of belonging were developed: role in a new social network, and social support and safety.

#### Role in a new social network

Many commented on the importance of creating a community and contributing to this community. Participants had different interpretations of what this contribution entailed. To some, it meant taking on responsibilities, like being a board member or on a party committee. While for others, it meant creating a pleasant environment by being social and taking initiative, by for instance inviting neighbours over for coffee or board games. It could also simply mean taking part in the social activities being arranged at the residency, regardless of a strong interest in that particular activity. When discussing the importance of creating a community, one participant said:

It doesn't happen by itself. You can't just sit around and wait. You have to say "hello" and invite people in for a cup of coffee or a glass of wine. It is something you must create yourself in order to make contacts and create a pleasant environment. Because for most people this is the final destination.

While some participated in almost everything being arranged, most attended 2–3 different activities a week. Having the ability to choose for themselves which activities they wanted to participate in and their level of social engagement seemed to have a positive effect on their overall level of satisfaction with the living environment. When discussing this topic one participant said:

Here you cannot go out without meeting someone, but you exchange a couple of phrases and then it is ok. You never feel people intrude. You can decide a lot for yourself here. There are also more options, and a lot friendlier compared to where we used to live.

Participants’ feelings of group affiliation were also reflected in the way they talked about the importance of helping others. This could for instance be by offering each other a ride to the city or inviting residents living alone over for small gatherings. Some also mentioned the moral commitment to include everyone, regardless of former relation. However, this emphasis on group affiliation made it especially apparent when someone did not find their place in the group. For the participants who expressed some level of dissatisfaction over the living situation, the social component was a central contributing factor. Some struggled to find likeminded people, while others were weighted by social conflicts that had occurred at the residency. As one participant explained:

There are some conflicts that have evolved here at the residency, in which people have ended up on different sides. This has also led to a lot of gossip. And I think it's sad, because I feel it is very much against the actual philosophy of the housing project.

#### Social support and safety

Being part of a group provided many with a feeling of social support and safety, making them less vulnerable compared to when they were living alone. Some mentioned the comfort and ease of being able to go alone to events at the residency, knowing you would be surrounded by people you know. This could be anything from casual movie nights to larger parties. As one participant explained:

It’s not dangerous to be alone here. For instance, if there is a party happening here, it's not dangerous to go up there alone and sit down. Because you kind of have everyone, you know.

For others, this feeling of social support was helpful when facing difficult times in life, like loneliness or depression. The support did not necessarily have to be explicit, just the feeling of having people around and being part of a social network could be enough. Some also mentioned the ease of venting to neighbours when having a troubled mind. One participant explained the difference of living in this environment compared to where she lived in the past:

You feel you have a network in a way. It has been much better for me to move up here compared to when I was in a poor housing association all by myself, because here there are people you can actually talk to. That is very important when you are depressed.

This feeling of social support and safety was also reflected in their experience with the COVID-19 pandemic, in which most of the participants felt relatively unaffected. Living in a controlled environment, with common infection prevention rules and knowledge of disease status among the residents, made most feel relaxed in regard to getting infected. On this topic, one participant said:

We have trusted each other, and everyone is careful and lets us know if they are infected and stays away. So, yes, it has been incredibly good.

Some also mentioned the comforting feeling of always having people around, thus preventing loneliness, a common consequence of social restrictions. As one participant explained:

At first you felt a bit isolated. But at the same time, we live in such a way that we always have people around, and that helps a lot.

## Discussion

### Summary of main findings

This study aimed to explore mechanisms important for the facilitation of active ageing in a community-based living environment in Norway. We developed three themes using RTA, involving the need for maintaining self-identity, experiencing growth and development, and feeling a sense of belonging. Maintaining self-identity concerned the ability to continue doing activities they valued or had enjoyed in the past, as well as having a role in life and maintaining their autonomy. Experiencing growth and development involved exchanging knowledge, learning new things, and experiencing mastery. Feeling a sense of belonging meant being part of a new social network and receiving social support. Concerning RQ1, most participants felt they had increased their social engagement and physical activity levels after moving to the living environment. This increase did not seem to relate to how active and social they perceived themselves originally. Instead, it seemed more related to having these core needs (themes) covered, making them important factors for facilitating active ageing in this living environment, answering RQ2 and demonstrating the importance of convenience in regard to participation and lifestyle changes.

Another interesting finding was the synergy effect that occurred within this community-based design in which the residents themselves became the resource: they *created* the learning arena by exchanging knowledge, *inspired* and motivated each other to be active and social, and *helped* each other when needed. Combined, this resulted in a self-sustaining environment in which the participants experienced both contribution and mastery whilst potentially reducing the need for external resources. Most were also minimally affected by the COVID-19 pandemic, suggesting community-based living could increase resilience among this age group. However, some social friction did occur at this residency. Although everyone appeared to be aware of the situation, there seemed to be little knowledge on how to solve the problem or where to put the responsibility. This is an example of a challenge with a self-sustaining model, in which the residents themselves are the board members dealing with conflict within their own environment. Moreover, what differentiates this from a typical apartment complex is how participatory the residents are, making it very visible when someone has not found their place in the group.

### Main findings in context

The results from this study share a lot of similarities from previous studies on similar housing environments, especially related to the environment providing a feeling of belonging and increased activity levels (8–10). In addition, the study on a housing community in Canada revealed similar challenges related to social dynamics (10). As with Helgetun, the residents experienced limited knowledge of conflict management, suggesting this may be an ongoing challenge with this type of living environment. Nonetheless, to our knowledge, no studies revealed similar findings regarding maintaining self-identity – although autonomy was mentioned in some of the studies. This may uncover a uniqueness with the design of Helgetun, which has a very flexible structure in which activities and opportunities are shaped by the interest and wishes of the residents, in a dynamic manner. This allows the residents to continue doing activities that are important to them, or they may have enjoyed in the past, contributing to them maintaining their self-identity.

To embrace the exploratory and inductive design of the study, I wanted to immerse myself in the setting with no prior theorical position. Meanwhile, during the development of the themes and discussions with the other co-authors, we were surprised how our findings turned out to align with relevant theories. For instance, the main themes draw close resemblance to the self-determination theory, concerning three basic psychological needs that must be satisfied to foster well-being and health, and allow for optimal function and growth (15). They involve the need for autonomy, competence, and relatedness. Studies conducted within this theoretical framework have shown promising results in other areas of health behavior change. For instance, research on physical activity motivation from the perspective of self-determination theory has grown considerably in recent years (16, 17). This research is based on the hypothesis that fulfilment of the three basic needs will intrinsically motivate people to participate in an activity. Indeed, a systematic review by Teixeira et al. found a positive correlation between self-determination theory-based exercise and exercise participation and long-term adherence (17). Studies have also found a positive correlation between self-determination theory and life satisfaction among different age groups in various contexts (18, 19).

Although studies have applied this theory in the context of behavioural change and well-being in general, less is known about how it can relate to the ageing population. This could be of particular interest seeing as the transition to later life can be challenging – with changes in occupational status, social life, and role in the family – and many struggle with developing new ways to fulfil their psychological needs. This corresponds well with our theme “maintaining self-identity” in which several participants elaborated on age-related changes in their sense of self and role in life. A book published in 2020 by Ng and Ho was the first to apply a framework of self-determination theory to analysis of healthy ageing (20). The authors hypothesized that in the context of healthy ageing, satisfying individuals’ basic psychological needs is likely to motivate individuals to participate in activities, thus promoting physical, social, and psychological well-being. They further encouraged future research to consider the nature of motivation among older adults via the self-determination framework (20). Our study provides empirical data on how optimizing the living environment to satisfy these needs may have a positive effect on residents’ activity levels and social engagement, thus facilitating active ageing and reducing the feeling of loneliness.

Although the need for autonomy, competence and relatedness are essential regardless of culture or life domain (15), the findings in our study are clearly shaped by Norwegian culture. Norway has a government-funded, rights-based healthcare system, and the majority of care-dependent older adults receive home care services or, if needed, are transferred to a nursing home. Consequently, people in Norway are not as dependent on family as seen in other countries and it is not general practice to live in inter-generational housings and take care of older family members (21–23). This was reflected in our findings, as many said they wanted to create meaningful lives for themselves, independent of family. There was an emphasis on not expecting their children to prioritize them, seeing as they now had their own life and burdens to deal with. Three of our authors (JC, BSH, MP), who hail from different nationalities than Norway, found these findings to be particularly interesting and different from their preconception of senior living. This attitude is perhaps more concentrated in the Nordic countries where the welfare state facilitates independence of the individual to a greater extent, as opposed to most other countries in the world. This implies that community-based living can be a particularly well-suited model for older adults living in Nordic countries, especially in the years to come when access to nursing homes and home-care services will be limited, and people are expected to plan for their own ageing.

### Future research and implications for practice

Our results demonstrate that providing older adults with the proper opportunities and environment can improve their physical activity levels and social engagement, regardless of their starting point. These factors are central for maintaining good health and well-being and preventing functional decline and disease development. Improving these factors can therefore have critical consequences for both the individual and society. On an individual level, it can lead to improved health outcomes and well-being, whilst on a societal level, it can reduce health costs and need for external resources.

Although this specific living environment (Helgetun) is expensive and not accessible for all, knowledge gained from it can be implemented into society to facilitate active ageing. Based on our findings, most of the gained benefits were related to the synergy effect that occurred when gathering a group of people in the same life situation. Thus, more focus should be put on creating accessible meeting arenas where people can exchange knowledge, learn new skills, inspire each other, and feel as a being part of a group. This can be implemented into existing neighbourhoods or be used as foundational pillars upon creating new living solutions for older adults. These findings are of key importance for older adults, politicians, stakeholders, and construction industries as people now are expected to live independently for longer and plan for their own ageing.

For future research it could be interesting to investigate how the community evolves over time: changes in social dynamics, how the community accommodate for residents eventually needing more care, how activity and socialization levels change over time, and finally, how the community adapts to new residents. The latter is particularly interesting, seeing how small and interconnected the community is, and since most of the residents in this case were part of the housing project from the beginning. It would also be interesting to uncover what motivates people to seek out these living environments, as people now must plan for their own ageing to a greater extent.

### Strengths and limitations of this study

Findings from this study provide comprehensive insights into how we should prepare and design for the future to facilitate active ageing, thus ensuring the health and well-being of the next generation of older adults. The study used an explorative approach with minimal pre-determined theoretical assumptions, and the findings were inductively developed through data collection and analysis, and subsequently related to theory. Another strength of this study is that it provides empirical data on how these community-based environments function compared to theory.

A limitation of the study is that the residents who volunteered to participate may not be a representative group for the entire residency. Similarly, the residents living in this environment had to apply and were selected to live there by the project developers themselves, and it may not be possible to implement knowledge gained from this living environment to society as a whole. For instance, all participants were white, Norwegian, cisgender individuals with an above average self-perceived financial status.

Regarding methodological implications, the relationship established between the researcher and participant may have prompted the interviews somewhat, seeing as some of the topics had already been discussed prior to the interview setting. This may have caused some degree of interview bias, in which the researcher asked leading questions based on existing knowledge about the participants. Nevertheless, in the context of this study, where data were collected from both the informal conversations and the arranged interviews, the weight of this bias is somewhat reduced.

## Conclusion

To successfully facilitate active ageing, community-based living environments should have a flexible structure adapting to the core needs of the residents. They should also embrace the resources the residents possess, resulting in a more self-sustaining environment in which residents experience both contribution and mastery whilst reducing the need for external resources. The results from this study contribute to the growing evidence that community-based living environments increase the residents’ social, mental, and physical participation, whilst reducing social isolation and loneliness, also in a Norwegian setting. These findings are meant as grounding points for policymakers to reflect upon designing future senior living spaces, potentially improving public health, and ensuring the well-being of older adults.

## Data availability statement

The datasets presented in this article are not readily available because the dataset generated from this study is not publicly available based on ethical considerations regarding recognition risk of the participants. It is also part of an ongoing doctoral research project containing unpublished data. Requests to access the datasets should be directed to elise.forsund@uib.no.

## Ethics statement

The studies involving humans were approved by Norsk senter for forskningsdata. The studies were conducted in accordance with the local legislation and institutional requirements. The participants provided their written informed consent to participate in this study.

## Author contributions

EF: Conceptualization, Data curation, Formal analysis, Investigation, Methodology, Resources, Software, Visualization, Writing – original draft, Writing – review & editing. JT: Conceptualization, Formal analysis, Methodology, Software, Supervision, Validation, Writing – original draft, Writing – review & editing. SF: Formal analysis, Methodology, Supervision, Writing – original draft, Writing – review & editing. HR: Conceptualization, Data curation, Project administration, Writing – review & editing. MP: Conceptualization, Project administration, Writing – review & editing. BH: Conceptualization, Funding acquisition, Project administration, Resources, Supervision, Validation, Writing – review & editing.

## References

[1] United Nations. 2022 revision of world population prospects D.o.E.a.S. Affairs and P Division (2022).

[2] Russell D Peplau LA Cutrona CE. The revised UCLA loneliness scale: concurrent and discriminant validity evidence. J Pers Soc Psychol. (1980) 39:472–80. doi: 10.1037/0022-3514.39.3.472, PMID: 7431205

[3] Bookman A. Innovative models of aging in place: transforming our communities for an aging population. Community Work Fam. (2008) 11:419–38. doi: 10.1080/13668800802362334

[4] Park JH Moon JH Kim HJ Kong MH Oh YH. Sedentary lifestyle: overview of updated evidence of potential health risks. Korean J Fam Med. (2020) 41:365–73. doi: 10.4082/kjfm.20.0165, PMID: 33242381 PMC7700832

[5] World Health Organization, Decade of healthy ageing: plan of action. (2020).

[6] World Health Organization. Active ageing: a policy framework. Geneva: World Health Organization (2002). 12040973

[7] Cummings S. Kropf N., Senior cohousing—history and theory. Springer. Book series: Springer Briefs in Aging (2020). p. 9–16.

[8] Rusinovic K Bochove MV Sande JV. Senior co-housing in the Netherlands: benefits and drawbacks for its residents. Int J Environ Res Public Health. (2019) 16:3776. doi: 10.3390/ijerph16193776, PMID: 31597278 PMC6801586

[9] Jolanki OH. Senior housing as a living environment that supports well-being in old age. Front Public Health. (2020) 8:589371. doi: 10.3389/fpubh.2020.589371 33614564 PMC7890193

[10] Puplampu V Matthews E Puplampu G Gross M Pathak S Peters S. The impact of cohousing on older Adults' quality of life. Can J Aging. (2020) 39:406–20. doi: 10.1017/S0714980819000448, PMID: 31422780

[11] Torrado JC Husebo BS Allore HG Erdal A Fæø SE Reithe H. Digital phenotyping by wearable-driven artificial intelligence in older adults and people with Parkinson's disease: protocol of the mixed method, cyclic ActiveAgeing study. PLoS One. (2022) 17:e0275747. doi: 10.1371/journal.pone.0275747, PMID: 36240173 PMC9565381

[12] Bunniss S Kelly DR. Research paradigms in medical education research. Med Educ. (2010) 44:358–66. doi: 10.1111/j.1365-2923.2009.03611.x 20444071

[13] Brewer J., Ethnography: understanding social research. (2004). University of Michigan. 312–322.

[14] Braun V Clarke V. Reflecting on reflexive thematic analysis. Qual Res Sport, Exerc Health. (2019) 11:589–97. doi: 10.1080/2159676X.2019.1628806

[15] Ryan RM Deci EL. Self-determination theory: Basic psychological needs in motivation, development, and wellness Guilford Publications (2018).

[16] Morgan GS Willmott M Ben-Shlomo Y Haase AM Campbell RM. A life fulfilled: positively influencing physical activity in older adults – a systematic review and meta-ethnography. BMC Public Health. (2019) 19:362. doi: 10.1186/s12889-019-6624-5, PMID: 30940111 PMC6444855

[17] Teixeira PJ Carraça EV Markland D Silva MN Ryan RM. Exercise, physical activity, and self-determination theory: a systematic review. Int J Behav Nutr Phys Act. (2012) 9:78. doi: 10.1186/1479-5868-9-78, PMID: 22726453 PMC3441783

[18] Stephan Y Fouquereau E Fernandez A. The relation between self-determination and retirement satisfaction among active retired individuals. Int J Aging Hum Dev. (2008) 66:329–45. doi: 10.2190/AG.66.4.d, PMID: 18507333

[19] Abu Daoud A Alkhateeb J. Life satisfaction and its relationship to self determination skills and Hope in adolescents with disabilities in Jordan. An-Najah Univ J Res B (Humanities). (2017) 31:1889–910. doi: 10.35552/0247-031-011-001

[20] Ng B Ho G. Self-determination theory and healthy aging: comparative contexts on physical and mental well-being. Singapore: Springer Nature. (2020).

[21] Andruske CL O'Connor D. Family care across diverse cultures: re-envisioning using a transnational lens. J Aging Stud. (2020) 55:100892. doi: 10.1016/j.jaging.2020.100892, PMID: 33272452 PMC7573693

[22] Lin J-P Yi C-C. Dilemmas of an aging society: family and state responsibilities for intergenerational Care in Taiwan. J Fam Issues. (2019) 40:1912–36. doi: 10.1177/0192513X19863204

[23] Rutagumirwa SK Hutter I Bailey A. “We never graduate from care giving roles”; cultural schemas for intergenerational care role among older adults in Tanzania. J Cross Cult Gerontol. (2020) 35:409–31. doi: 10.1007/s10823-020-09412-w, PMID: 32990906

